# “Feels Good to Get Wet”: The Unique Affordances of Surf Therapy Among Australian Youth

**DOI:** 10.3389/fpsyg.2021.721238

**Published:** 2021-10-14

**Authors:** Rebekah Jade McKenzie, Timothy P. Chambers, Kathryn Nicholson-Perry, Joel Pilgrim, Philip B. Ward

**Affiliations:** ^1^Discipline of Psychological Science, Australian College of Applied Psychology, Sydney, NSW, Australia; ^2^School of Psychology, Deakin University, Melbourne, VIC, Australia; ^3^Waves of Wellness Foundation, Sydney, NSW, Australia; ^4^UNSW Sydney and Schizophrenia Research Unit, School of Psychiatry, Ingham Institute for Applied Medical Research, Sydney, NSW, Australia

**Keywords:** mental health, ecological dynamics perspective, exercise, nature, surf therapy, adolescence

## Abstract

Increasing prevalence rates of mental health problems among adolescents is an issue of growing concern. Surf therapy is a novel intervention that may provide tangible benefits to address this problem. Congruent with the ecological dynamics perspective (EDP), the existing research postulates that surf therapy yields psychological benefits in part due to the unique affordances of immersion in a blue space such as the ocean. Evidence worldwide has validated the use of surfing as a therapeutic mediator to achieve positive change, however, no such research has been conducted on Australian adolescents. The current study aimed to evaluate the impact of the Waves of Wellness (WOW) Foundation's 8 week surf therapy program on mental health outcomes in Australian at-risk youth. The study employed a mixed-methods design to examine the experiences of participants. Nine adolescents (*M*_*age*_ = 14.9; *SD* = 1.2; 8 female) participated in the quantitative stage, with four completing the qualitative interview. Results indicated positive changes in resilience, self-esteem, social connectedness, and depressive symptoms post-intervention, however, improvements were not maintained at follow-up. Participants unanimously agreed that the program normalised their experiences with mental health through the unique leaning environment and fostered personal growth through mastery experiences and the development of healthy relationships. The EDP provided the most compelling explanation of the results denoting that benefits arise from the reciprocal relationship between individual, task, and environment. Despite the small and heterogenous sample, the findings provided preliminary evidence of the efficacy of surf therapy among Australian youth and offer a potential starting point for further research utilising larger more diverse samples.

The increasing prevalence of mental health problems among adolescents is an issue of growing concern. Half of all mental health disorders begin before the age of 14 years, with 10–20% of adolescents experiencing mental health conditions globally (Kessler et al., 2007). In Australia, one in seven (14%) children and adolescents had a mental disorder in the previous 12 months; for those aged 12–17, over 11% engaged in self-harming behaviours and 6% had thoughts of suicide (Australian Institute of Health Welfare, 2016). These statistics highlight the importance of psychological well-being among young people, and the need to provide engaging and accessible services to improve well-being, including interventions that increase coping strategies in order to manage daily stressors and successfully transition into adulthood (Bandura, 2000).

It is well-established that regular physical activity can enhance mood and alleviate mental health issues (Rosenbaum et al., 2014; Firth et al., 2019). Aerobic exercises such as jogging, swimming, cycling, walking, surfing, and dancing have all been shown to reduce symptoms of anxiety and depression (Guszkowska, 2004), improve mood and lessen cognitive decline (Babyak et al., 2000). Similarly, a systematic review and meta-analysis utilising longitudinal and cross-sectional studies found significant relationships between exercise and lower levels of mental health symptoms, and greater psychological well-being for youth (Rodriguez-Ayllon et al., 2019); there appears a causal association between exercise and cognitive functioning and a partial causal association between exercise and depression for this cohort (Schuch et al., 2018; Biddle et al., 2019). Accordingly, attention has turned to examining exercise context, with a particular focus on nature-based exercise.

Early research regarding exercise in natural environments has revealed that individuals experience greater reductions in anger and depression and significant increases in positive self-reported emotions compared with exercise in synthetic environments such as gyms or urban landscapes (Bowler et al., 2010; Thompson Coon et al., 2011). However, these findings were criticised by the authors because of the poor quality of available research due to methodological limitations and heterogeneity of outcome measures (Thompson Coon et al., 2011).

One environmental context that has received less attention is water-based physical activity, with researchers suggesting that swimming and other water-based activities (e.g., kayaking, surfing) may provide superior benefits for the promotion of human well-being due to greater affordances (Foley and Kistemann, 2015; Araujo et al., 2019). These activities have heightened the psychological benefits (e.g., reduced anxiety, depression, and perceived stress, increased positive affect, and increased quality of life) associated with exercise and nature among US war veterans (Lundberg et al., 2011; Bennett et al., 2017; Walter et al., 2019). Further, there is increasing evidence suggesting that participation in action and adventure sports, such as surfing, is meaningful and life-enhancing (Immonen et al., 2017). Participating in action and adventure sports is said to create opportunities to foster positive psychological outcomes such as resilience, self-esteem, positive affect, and relatedness (Brymer and Gray, 2009; Brymer and Schweitzer, 2013; Clough et al., 2016). A growing body of evidence worldwide has validated the use of surfing as a therapeutic mediator to achieve positive change such as increased well-being (de Matos et al., 2017). Surf therapy is defined by the International Surf Therapy Organisation (International Surf Therapy Organization, 2019) as an intervention that combines surf instruction, surfing, and structured individual and/or group activities to promote physical, psychosocial, and psychological well-being. In addition to physical exercise, surf therapy interventions typically include individual mentoring, social skills development, psychoeducation, and group discussions focused on increasing resilience and personal growth (Benninger et al., 2020). Current programs can be found throughout the world and vary in their structure, duration, and content (see International Surf Therapy Organization, 2020). Surfing affords the opportunity to combine the psychological benefits of socialising, nature, and exercise by participating in water-based exercise (Fleischmann et al., 2011).

The emerging evidence-base suggests that surf therapy is effective in improving mental health outcomes among vulnerable youth, disabled populations, and military veterans (Fleischmann et al., 2011; Godfrey et al., 2015). For example, a study conducted in the United Kingdom (UK) examining the effects of surfing on the well-being of combat veterans found that surfing elevated subjective well-being through a sense of respite from worries, increased positive emotions, and connection with others (Caddick et al., 2015). Surf therapy has also been found to be associated with significantly lower post-traumatic stress disorder and depressive symptoms (at both the conclusion of therapy and 30 day follow up), increases in positive affect, and decrease negative affect, anxiety, and depression (Rogers et al., 2014; Crawford, 2016; Walter et al., 2019). A review of the existing literature on surf therapy concluded that for youth in need of psychological support, outcomes following surf interventions included increases in self-concept, emotion regulation, social competencies, and social connections, as well as decreases in behavioural problems and depressive symptoms (Benninger et al., 2020). These studies provide provisional support for the use of surf therapy as an effective intervention among adolescent populations to address concerns regarding mental health and well-being.

Despite the growing body of literature that demonstrates the efficacy of surf therapy in improving mental health, few studies have examined the mechanisms by which surf therapy impacts well-being and mental health. Marshall et al.'s (2019) study examining the underlying mechanisms of the effects of surf therapy on mental health concluded that two core components were fundamental to positive outcomes: self-selected progress and the creation of an emotional and physical safe space. They posited that different levels of difficulty within the waves, removal of perceptions of failure within the group, and increased support from peers and mentors facilitated the achievement of these core components. Moreover, these core components facilitate improvements in mental health through a sense of mastery and achievement from learning a new skill, a sense of respite or escape, and increased social connections with their peers (Marshall et al., 2019).

When considering the theoretical underpinnings of such research, there are four prominent theories that explain the benefits of nature-based activities including: Attention Restoration Theory (ART; Kaplan and Kaplan, 1989), Stress Reduction Theory (SRT; Ulrich, 1993), Biophilia Theory (Wilson, 1984; Kellert and Wilson, 1993), and the Ecological Dynamics Perspective (EDP; Brymer and Davids, 2013). Whilst ART (Kaplan and Kaplan, 1989), SRT (Ulrich, 1993), and the Biophilia Theory (Wilson, 1984; Kellert and Wilson, 1993) are robust in their own right, these theories have attracted criticism for exclusively focusing on the individual (i.e., organism) and have neglected investigating the role of the environment. Unlike these theories, EDP is a holistic perspective of behaviour that seeks to explain how individuals form processes of action, perception, and cognition by taking into consideration the interactions of the individual, environment, and task (Brymer and Davids, 2013). EDP highlights the importance of the interactive relationship between an individual and the environment in the explanation of individual differences in learning and behavioural change (Brymer and Davids, 2013). It is a theoretical framework that views individuals as composed of many distinct but interacting systems (Chow et al., 2011). EDP therefore proposes that the psychological benefits of nature, including the performance of action and adventure sports in this context, may be a function of the interactive relationship between the individual, their unique characteristics and experiences, the sport (i.e., surfing), and the environment. Critical to the EDP is the notion of affordances. Affordances are conceptualised as the series of functionally significant properties within an environment that are meaningful to the individual, which thereby enable the individual to develop a relationship with the environment by inviting them to perform a variety of behaviours (Araujo et al., 2019). For example, an individual might surf on, splash in, dive through, or even watch ocean waves, such that one wave can represent a multitude of affordances for different individuals.

Congruent with the EDP, the existing research postulates that surf therapy yields psychological benefits due to the unique affordances of immersion in a ubiquitous environment such as the ocean. It is postulated that surfing allows for a heightened sensory experience (Godfrey et al., 2015), facilitates respite, builds self-efficacy (Fleischmann et al., 2011), promotes a sense of mastery, and fosters a culture of acceptance and connection (Godfrey et al., 2015; Marshall et al., 2019). Previous research has found that athletes experience the natural world as a facilitator of a profound and positive understanding of self and one's place in the environment (Brymer and Gray, 2009). These proposed mechanisms collectively emphasise the interactive nature of the relationship between task (e.g., surfing), individual (participant), and environment (ocean), reiterating the foundation of the EDP, and highlighting the utility of surf therapy among at-risk youth.

## The Current Study

The preliminary surf therapy research implies that there are many tangible benefits for participants undergoing this form of intervention (Benninger et al., 2020) and the high prevalence of surfing and accessibility to conducive conditions in Australia makes this a promising area of inquiry. However, the current evidence-base for surf theory is limited. Whilst there are some empirical investigations that serve as a platform for this study, there is no evidence related to the effect of surf therapy on Australian adolescents. Further, given the prevalence of mental health concerns for this population, there is a strong need to investigate alternate interventions that may be of assistance.

The general aim of the current study is to examine the effects and perceived benefits of participating in an 8 week surf therapy program provided by the Waves of Wellness Foundation (Waves of Wellness Foundation, 2020) on psychological outcomes among Australian at-risk youth. For the purpose of the current study, at-risk youth were defined as young people at greater risk or vulnerability for mental health disorders, such as depression and anxiety, or problem behaviours, such as social isolation, substance abuse, or school refusal and/or failure (Lubans et al., 2012). Based on the current evidence base, the study examined the effects of the WOW program on resilience, self-esteem, social connectedness, and negative psychological symptoms. As an exploratory study, the specific aims were to:

1. Advance current understandings of surf therapy as an alternative therapy for youth populations.
2. Inform future research by examining participants' experiences of the program.

## Methodology

### Theoretical Framework and Design

The current research adopted an interpretive paradigm with an assumption of ontological relativism and epistemological constructionism (Crotty, 1998; Guba and Lincoln, 2005). This philosophical position aligns with the understanding that reality is subjective, differing between individuals. Knowledge and meaning are co-constructed through the interaction of the researcher (separate from the experience) and the participant who each influence issues through their own experiences and understandings (Poucher et al., 2020). The aim of examining participants' experiences of the WOW program is consistent with the interpretive paradigm which aims to understand individual meanings created from experiences (Poucher et al., 2020). Therefore, a mixed method approach was employed, allowing for the integration of data and a holistic understanding of individual experiences. Mixed-methods (semi-structured interviews and trend analysis) was deemed most suitable for the current study as it enabled quantitative findings to be grounded in participants' experiences, better capturing the voice of youth in the research.

### Participants

The current study was conducted within the August 2020 WOW surf therapy program on Bondi Beach in Sydney, Australia. In total, nine Australian youth participated in the quantitative stage. Participants were aged between 14 and 17 years (*M*_*age*_ = 14.9; *SD* = 1.2), predominately female (*n* = 8), and identified as either Caucasian (*n* = 6), Asian (*n* = 1), European (*n* = 1), or Aboriginal (*n* = 1). Table 1 shows demographics collected pertaining to pre-existing mental health- related diagnoses and sports experience. For the qualitative evaluations, four of the original nine participants volunteered to participate in semi-structured interviews.

**Table 1:** Demographic information for participants (*n* = 9).

**Participant**	**Gender**	**Age**	**Diagnoses**	**Sport**	**Previous frequency of sport a week**	**Current frequency of sport a week**
P1	F	14	ADD	Netball/soccer/swimming/ gymnastics/sailing/surfing/ volleyball/kayaking	2–3	2–3
P2^*^	F	14	N/A	Surfing/netball/soccer/ cricket/swimming	4–6	2–3
P3^*^	M	14	N/A	Surfing/AFL/athletics	7	7
P4^*^	F	17	N/A	N/A	<1	1
P5^*^	F	15	OCD/Anxiety/Depression	Ice hockey/ soccer/running/netball	1	2–3
P6	F	16	N/A	Rhythmic gymnastics	2–3	4–6
P7	F	16	N/A	Gym	2–3	4–6
P8	F	14	N/A	Football/netball/basketball	1	1
P9	F	14	Anxiety	Touch football/ soccer/netball/surf life saving	2–3	1

* *Participants who agreed to be interviewed.*

*F, Female; M, Male; OCD, Obsessive Compulsive Disorder; ADD, Attention Deficit Disorder*.

Participants for this program were recruited through promotion with WOW's partner mental health organisations in Sydney, including headspace Bondi Junction. Youth from the general public were also able to access the program via referral from their general practitioner and or case manager, along with self-referral. All individuals voluntarily consented to take part in the research.

The inclusion criteria for the current study required participants to be aged 14–17 years old. No other inclusion criteria were applied for the following reasons: (1) to align with WOW's mission of providing accessible and innovative mental health treatment and support by empowering individuals with tools for wellness and strengthening community connection, and (2) ethical approval was not granted to target a specific clinical sample. As such, WOW accepted the first 10 participants who met the broad selection criteria into the program.

### Intervention

**Figure 1:**
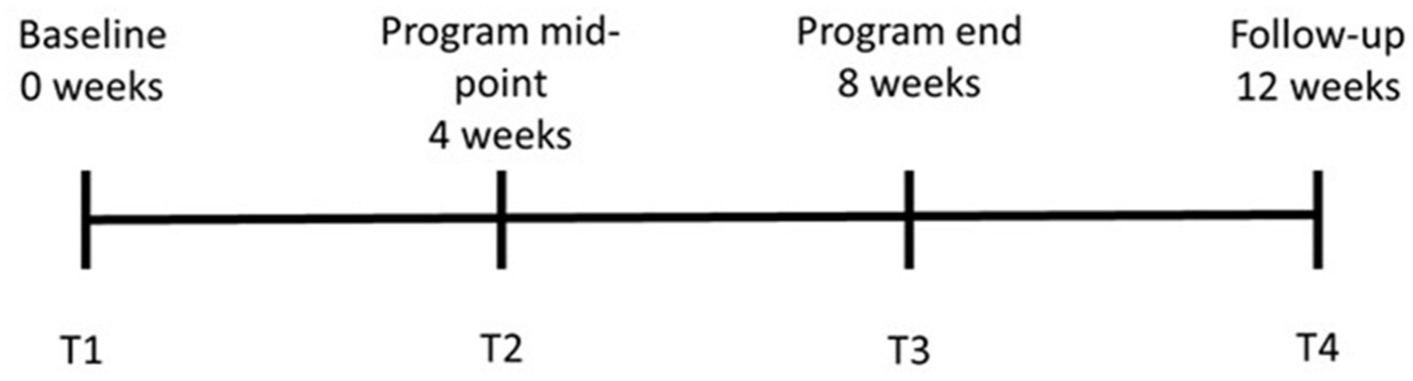
Program lifespan and critical time-points.

The WOW surf experience program consisted of weekly 2 h early morning sessions for 8 weeks. Each session involved a 45 min discussion related to mental health on the sand, followed by 60 min in the ocean surfing. The sessions were facilitated by mental health clinicians who were qualified surf instructors at a ratio of one clinician per five participants, with the group size limited to 10 participants. Discussion topics for the 8 weeks of the program included: physical and mental wellness, identifying healthy and unhealthy emotions, managing change, mindfulness, problem solving and asking for help, relationships, meaning making, and looking to the future. Figure 1 illustrates the various research activities that were scheduled across the program lifespan.

### Materials

#### Quantitative Measures

Four self-report measures captured the constructs to be measured: depression, resilience, self-esteem, and social connectedness. Participants were also required to complete an eight-item demographic questionnaire pertaining to their age, gender, ethnicity, diagnosis, work/study status, current, and past engagement in sports and exercise. All measures were completed online *via* Qualtrics at T1, T2, T3, and T4 with the exception of the demographic information (T1 only), as shown in Figure 1.

##### Rosenberg Self-Esteem Scale (RSES)

Participants reported how they generally feel about themselves on ten items (five positively worded and five negatively worded items) using a 1 (“strongly disagree”) to 4 (“strongly agree”) scale. Scores were derived using standard scoring procedures (Rosenberg, 1965), with greater scores indicating higher self-esteem. Reliability analyses indicated evidence of internal consistency (α = 0.93), and test-rest reliability (*r* = 0.85; Koruklu, 2015).

##### Patient Health Questionnaire for Adolescents (PHQ-A)

Negative psychological symptoms were assessed via the PHQ-A; participants reported how bothered they have been in the past 2 weeks on nine symptoms of depression using a 1 (“not at all”) to 4 (“nearly every day”) scale. They also reported the frequency of depressive symptoms over the past year, functional impact of these difficulties, thoughts of suicide, and previous suicide attempts. Scores were derived using standard scoring procedures (Johnson et al., 2002). The PHQ-A has been established to have good psychometric properties such as good internal consistency (α = 0.88), re-test reliability (*r* = 0.83), diagnostic validity, and moderate to good convergent validity (Nandakumar et al., 2018).

##### Brief Resilience Scale (BRS)

Participants reported how quickly they bounced back or recovered from stress on six items (three positively worded and three negatively worded items) using a 1 (“strongly disagree”) to 5 (“strongly agree”) scale. Scores were derived using standard scoring procedures (Smith et al., 2008), with higher scores indicating greater resilience. Evidence suggests good internal consistency (α = 0.80–0.91), re-test reliability (*r* = 0.61–0.69), convergent validity, and discriminant validity (Smith et al., 2008).

##### Social Connectedness Scale-Revised (SCS-R)

The 20-item index is a validated measure of social connectedness (Lee et al., 2001). Participants reported how they view themselves on 20 items (10 positively worded and 10 negatively worded items) using a 1 (“strongly disagree”) to 6 (“strongly agree”) scale. Responses were scored based on standard scoring procedures (Lee et al., 2001), with higher scores indicate a greater sense of connectedness. The SCS-R has been established to have good internal consistency (α = 0.92), convergent validity, and discriminant validity (Lee et al., 2001).

#### Qualitative Measures

A semi-structured interview schedule was created that centred on a set of open-ended questions to ensure greater interview flexibility (DiCicco-Bloom and Crabtree, 2006). Participants were encouraged to elaborate on all aspects of their experience and an opportunity was provided at the end of the interview to add any further information not already addressed.

### Procedure

The research was conducted in a community-based charity funded program for adolescents. Ethical approval was granted by the Navitas Professional Humans Research and Ethics Committee (558050220). In accordance with ethical approval, an information pack including an explanatory statement and an informed consent form was provided to WOW who then emailed the information to the parents and/or guardians of prospective surf therapy participants. At the beginning of the first session (i.e., after obtaining parental/guardian consent), participants were provided with the survey on Qualtrics using a clinician's mobile device and asked to register electronically. Subsequently, participants completed the 10 min survey package (T1). The survey measures were completed in the order as presented above.

Participants also completed the survey at weeks 4 (T2) and 8 (T3) immediately after the conclusion of the 2 h WOW session. They then completed the survey again at the 4 week follow up (T4). Semi-structured interviews were conducted at T4 by the first author and included questions pertaining to the participants experience of the program (e.g., what it taught them, was it a positive or negative experience), the impact of the program on their life (e.g., did it change anything about them such as mental health or social connection), the aspects of the program they enjoyed or felt they benefit from, and any general comments or observations they wished to share regarding the program and its' impacts. Due to COVID-19 related restrictions imposed by the authors' ethics committee all interviews were conducted *via* Zoom. All interviews were audio recorded and lasted between 10 and 18 min (*M* = 14.0, *SD* = 3.3). Participants were awarded a $20 gift-card for completing surveys and an additional $20 gift card for completing an interview.

### Data Analysis

#### Quantitative Analysis

Participants' results from pre, mid, post, and follow-up measures were analysed using Statistical Package for Social Sciences 27.0 (SPSS). It is worth noting that as originally planned the study would have utilised growth curve modelling to examine both similarities and differences among individuals (Frey, 2018). Unfortunately, due to COVID-19 restrictions recruitment was low resulting in a small sample size and inadequate power. Therefore, the focus of the data analysis shifted to examining the relationship between the quantitative and qualitative data utilising visual analysis of graphs and descriptive statistics.

#### Qualitative Analysis

Interviews were analysed by the first author using contemporary reflexive thematic analysis practises (Braun et al., 2016; Braun and Clarke, 2019). Congruent with our relativist approach, reflexive thematic analysis was utilised to identify, analyse, and report patterns of participant's opinions and experiences. Subsequent to transcription of interviews, transcripts were read and re-read by the first author to allow for familiarisation with the data. The coding phase began by hand whereby relevant data was highlighted to capture relevant concepts. Succinct initial codes were generated from relevant concepts that led to the deductive creation of latent themes. A thematic map was utilised to make sense of interconnections between codes and themes to further refine them into lower order and higher order themes. Themes were constructed coherently with research questions allowing for decisions to be made regarding relevant data segments for themes. Themes were defined following a review of their distinctiveness. Continuation of the analysis involved selecting relevant quotations to represent themes selected.

#### Methodological Rigour

To ensure trustworthiness of data and rigour in the research process (Smith and McGannon, 2018) several methods consistent with the ontological position and relevant to research aims were employed. It is noteworthy that thematic analysis acknowledges the reliance on the researcher's judgement and experiences in co-constructing knowledge with participants subsequently influencing analyses and conclusions (Poucher et al., 2020). Therefore, researchers reflected upon their role in the study, including experience and interpretations, to ensure the data were not obscured. The first author at the time of the study was an active mental health practitioner for adolescents, which served as a strength of the research process as it allowed for rapport to be created during interviews through use of age-appropriate language and interactions. The second author, an experienced qualitative researcher in the sport and exercise psychology field, served the role of critical friend. As a critical friend, the second author provided feedback and offered possible alternate interpretations of the data which assisted in the creation of themes (Smith and McGannon, 2018). Member reflections were also utilised whereby participants were invited to reflect on their experiences shared during the interviews and expand upon any detail by offering further insight (Smith and McGannon, 2018). Participants were asked to reflect on their transcripts and with the researcher's aid provided further explanations and opinions to generate additional data (Schinke et al., 2013). Lastly, quotations are provided in the results to support interpretations of data.

## Results

### Quantitative Results

**Figure 2:**
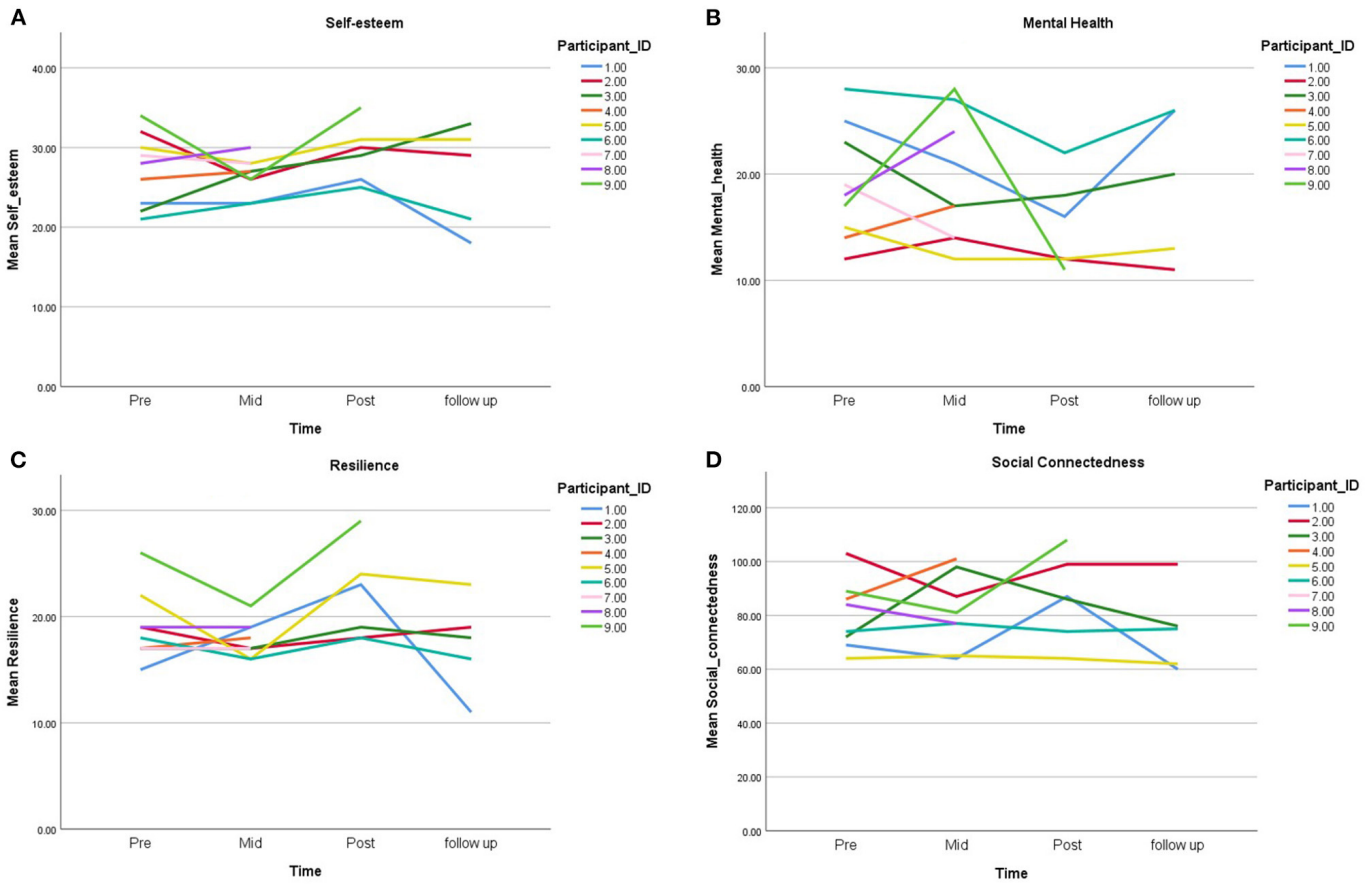
Participant scores of self-report measures as a function of time: **(A)** self-esteem, **(B)** mental health, **(C)** resilience, and **(D)** social connectedness.

Nine participants completed pre and mid-treatment measures, six participants completed pre-, mid-, and post-treatment measures, and five participants completed pre, mid, post, and follow-up measures. Figure 2 displays participants' scores for all measures pre, mid, post, and follow-up treatment.

Examination of descriptive statistics revealed a consistent pattern across variables from pre-intervention to follow-up. Mean changes on all measures indicated improvements from pre- to post-treatment, however, these improvements were not maintained at follow-up. A range of inferential test options were considered and explored (e.g., growth curve modelling, paired sample *t*-tests, correlations, and within-subjects ANOVA). Due to difficulties in recruitment and subsequent lack of power, a decision was made to not report or discuss any inferential results.

### Qualitative Results

**Figure 3:**
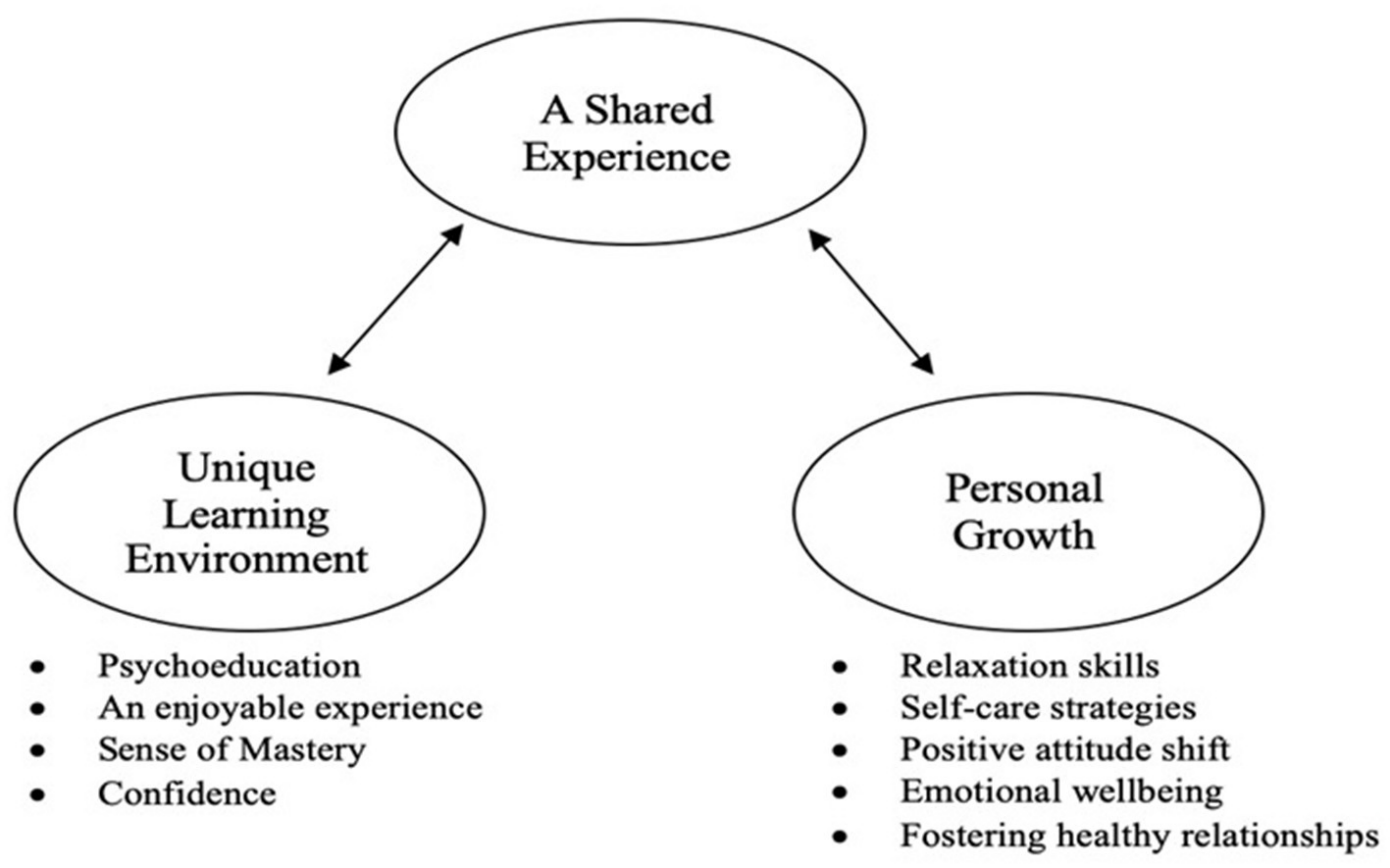
Thematic map of participant experiences in the WOW surf program.

The thematic analysis resulted in the construction of one higher order theme: a shared experience. As the label suggests, this theme explains participants' collective experiences during the surf therapy, and it encapsulated two lower order themes: (a) a unique learning environment and (b) personal growth (see Figure 3). Participants collectively agreed that the program enabled them to normalise their struggles with mental health and shift their perceptions that they were suffering alone. The water-based environment facilitated deeper understandings of mental health, acquisition of coping strategies, and fostered mastery experiences. Participants reported that the environment and enjoyment of the program resulted in noticeable improvements in emotional well-being, attitudes, and confidence. Finally, the experiential nature of the program was noted to help establish the importance of relaxation and self-care and demonstrated how to foster healthy relationships.

The following sections provide further description of these identified themes utilising quotations elicited from participants.

#### A Shared Experience

The higher order theme reflected the participant's general experiences throughout the program. In exploring participants perceptions of the program, it was unanimous that the program was enjoyable and ultimately enabled participants to normalise their mental health experience. The most commonly perceived benefit of the program identified by participants was the realisation that they are not alone or different, as many individuals struggle with mental health similarly to them: “it made me feel less alone and realise that there are lots of other people who feel the same way I do and are going through similar struggles.” (P5) Similarly, P4 stated: “everyone goes through the [tough] times and it's common. It's not only you. Just have to realise that.” Participants came to realise that the program enabled them to confidently share their personal experiences which aided them in putting their own difficulties with mental health into perspective. As P3 stated:

Lots of people are going through harder things, and everyone kind of has a relatable thing that comes in and you know you're taking everything for granted … you see that you're not the only one that's going through something.

Whilst not explicitly stated, it became apparent during the interviews that participants had shifted their perceptions related to their mental health concerns; the previously held belief that each individual experienced unique challenges was replaced with a shared appreciation that they were not alone in experiencing mental health issues. The open discussions during the WOW program appeared to also reduce the stigma associated with mental health difficulties and increased participants' empathy.

##### Unique Learning Environment

Collectively, the participants agreed that the program was effective in providing an environment that facilitated psychoeducation, an enjoyable experience, a sense of mastery, and increased confidence. Participants noted that the program enabled a deeper understanding of mental health and aided them in acquiring therapeutic strategies beneficial in coping with difficult experiences. For example, P4 stated: “it tells me that its ok like feeling down or like feeling like not as great as other times but like that's how life is and that's a big lesson I've learnt from WOW.” Similarly, P5 stated: “it was very helpful in learning certain tools.” Participants recognised that the program provided them with a more accurate understanding of changing emotional states and psychological tools which aided in coping with the range of moods experienced in adolescence.

The program was also deemed an enjoyable experience, as described by P2: “it was a pretty positive experience, it was very fun” and P5: “I really enjoyed the meditation and just going out on the water, it made me like feel the best in the mornings and it gave me something to look forward to every week.” Whilst not explicitly stated, it became apparent during the interviews that the participants felt the program offered a non-judgemental and supportive environment and removed any perceptions of failure among participants. As a result, participants were able to enjoy the experience without fear of judgement and unanimously agreed that the ocean, the social environment, and the facilitators' positive attitudes were responsible for their positive experiences.

The physical act of surfing presented the opportunity for participants to engage in mastery experiences during the program. Participants reflected that when they learnt a new skill or successfully performed it, they felt a sense of achievement and mastery which they noted positively impacted their mood and confidence. P3 stated: “it felt great to like progress you know. That's the best feeling, getting better at something.” Similarly, P2 stated:

“at least with the surfing it was like I had achieved something that day and I felt like more like it wasn't like a bad day and also more like happy positive attitude from like achieving that and being happy carried out through the majority of the day.”

Participants further reported that they felt more confident following the program as described by P4: “I think it does give me quite a lot of confidence.” These mastery experiences provided participants with the opportunity to persevere and believe in their own abilities to succeed which they collectively agreed had positive impacts on their confidence and emotional states.

##### Personal Growth

Participants agreed that they felt they had changed and developed as a result of the program. Participants reported that through engaging in the program they noticed development of relaxation skills, self-care strategies, positive attitude shifts, improved emotional well-being, and the ability to foster healthy relationships. Participants reported that the program increased their awareness of the need for self-care and aided the development of relaxation skills and self-care strategies. For example, P4 stated: “it also can help me watching my mental health a bit more and take care of myself more.” Similarly, P5 stated:

“it taught me that there are ways to like relax and unwind and distract myself umm when things get hard. Everyone shared different strategies they used when they weren't feeling the best. After that conversation I was more likely to think about going on a walk when I was stressed.”

This suggests that although participants had some elements of relaxation and self-care already embedded in their lives, the program helped to highlight their importance and provided opportunity for reflection on current practises facilitating personal growth in these areas.

In addition to growth in personal practises, participants reported that they experienced a positive shift in their attitudes towards daily activities and annoyances and greater emotional well-being, particularly on days where they engaged in the program. They reported increases in tolerance, willingness, concentration, alertness, and motivation as a result of the program. Further, they described emotional states congruent with well-being such as increases in happiness and satisfaction. P3 stated: “you felt more alive. I felt like I could concentrate a lot better at school and in general and it was just a better day” and P4 stated: “when you surf you just yell and everyone just kind of gets so happy.” Whilst not explicitly stated these outcomes appeared to be a result of the freedom felt from being in the water in a non-judgemental environment, allowing participants to enjoy respite from their daily worries.

Finally, the experiential nature of the program allowed participants to cultivate relationships with each other promoting the development and practise of fostering connections with others. For example, P4 stated: “I think surfing also makes us bond together more and feel more connected.” Topics of discussions specifically aimed at identifying what constitutes a health relationship also prompted reflection among participants about their own relationships. Combined these experiences enabled personal growth in this area through a shift in perspective from trying to control relationships to accepting relationships and enjoying them for what they are. P5 stated:

“Everyone in the group shared their personal experiences and gave insight into the relationships in their lives and the importance, or in some cases, detriment of them. I personally came to the conclusion to not take things so seriously, that friends will come and go, and to simply enjoy the moments with the people I love.”

Overall, the shared experience in a unique learning environment allowed participants to engage in learning and personal growth across a range of areas. These results suggested that surf therapy may afford distinct opportunities and benefits not found in traditional therapies which are well-suited to this population.

## Discussion

The current study was, to the best of our knowledge, the first study to examine the impact of surf therapy on Australian at-risk youth. The aim was to examine the impact of the WOW 8 week surf therapy program among Australian at-risk youth between 14 and 17 years old. A mixed methods research design was adopted to examine the experiences of participants and effects of the WOW program on self-esteem, resilience, social connectedness, and depressive symptoms. The WOW 8 week surf therapy program was implemented in Bondi, Sydney, with nine participants. The sessions combined the action of surfing with psychoeducational discussions aimed at increasing awareness and understanding of various aspects of mental health.

Quantitative findings provided evidence of positive change in self-esteem, resilience, social connectedness, and depressive symptoms after 8 weeks of surf therapy. Moreover, qualitative findings provided unique insights into participant's experiences, thoughts, and attitudes towards the program allowing for a deeper understanding of patterns found in quantitative results. Themes highlighted the natural affordances of surf therapy including the unique learning opportunities it provides allowing for personal growth among youth. Examining the underlying processes, the program influenced a number of psychosocial variables that have previously been implicated in increasing mental health among adolescents, including facilitating mastery experiences, normalising thoughts and feelings, and strengthening connection with others (Marshall et al., 2019). These results are consistent with similar research and the EDP model.

In line with previous research from various countries (Godfrey et al., 2015; de Matos et al., 2017; Marshall et al., 2019), the current study documented that surf therapy can increase social and emotional competencies among adolescent populations through building confidence, promoting a sense of mastery, and fostering a culture of acceptance and connection. These findings are in agreeance with de Matos et al. (2017) who concluded that surf therapy is beneficial for promoting well-being, emotional regulation, and personal and social skills in vulnerable youth populations, as well as, Godfrey et al. (2015) and Marshall et al. (2019) who reported participant experiences including resilience, self-esteem, friendship, happiness, and a sense of achievement or mastery. Moreover, there are demonstratable alignments between Yalom's therapeutic factors for group psychotherapy (Hauber et al., 2019) and the qualitative findings, including cohesion (i.e., participants were accepting of one another), guidance (i.e., sharing of psychoeducation resources), universality (i.e., the recognition that other participants had similar experiences), and self-understanding (i.e., learning to recognise that participants can exert control over their lives). Therefore, surf therapy seems effective in producing a myriad of benefits on mental health among this population.

When interpreting the findings, three explanations are evident. First, the seeming success of this intervention can be attributed to the unique affordances of the being immersed in water. We postulate that the heightened sensory experience of the ocean enables individuals to be engaged in the present moment creating a sense of respite and allowing for intense focus, enjoyment, and a sense of intrinsic reward (Godfrey et al., 2015). Second, the WOW surf program fosters mastery experiences thus bolstering self-esteem and resilience. That is setting a goal, persisting through challenges, and enjoying one's success increases the belief in ones' ability own to succeed. These mastery experiences provide evidence to suggest that the WOW program is distinctively suited to addressing mental health difficulties in this population. Third, the experiential nature of the program encourages a sense of connection through contact with participants and facilitators promoting the ability to develop positive relationships.

Unlike previous studies, however, the current study was unable to find statistically significant changes in these variables from pre to post-intervention. It is postulated that the small sample sizes in the current study are responsible for the lack of statistical significance. Moreover, the conditions of the natural environment (e.g., water/air temperature) may have contributed to participants' self-reported experiences, as previous research has demonstrated that extreme environments can affect individual mood state (e.g., Lane et al., 2004). Nonetheless, congruent with Godfrey et al. (2015) current findings provided preliminary support for the use of surf therapy as an appropriate and cost-effective mental health care option for adolescents and extended these findings to an Australian context.

Congruent with the EDP (Brymer and Davids, 2013) the current research further established that benefits arise from the reciprocal relationship between individual, task, and environment. The unique affordances of the blue environment combined with the practise of surfing allowed for personal growth and improvements in mental health. This was true even for participants who had previous experience with surfing. It is postulated that the social aspects of the program, such as discussions of personal experiences, contributed to the unique environment and facilitated positive improvements even among participants who regularly engage in surfing outside of the program. These results, in line with the EDP model, suggest that regular consistent exposure to nature may be needed in order to experience the benefits of such environment by maintaining the person-environment relationship.

Consistent with Araujo et al. (2019) the current study is consistent with the notion that EDP provides the most compelling explanation of the added benefits of exercising in a ubiquitous environment. The unique learning environment of the ocean demands holistic involvement of participants while surfing demands cognitive and emotional attention. Further, the affordances of the social interactions provide opportunities to develop relationships and confidence in ones' abilities. Combined these affordances of nature, surfing, and social interaction lead to personal growth through overcoming challenges and developing the necessary knowledge and confidence to do so again in the future.

## Limitations

The current mixed methods study provided preliminary evidence of the efficacy of surf therapy in improving elements of mental health in Australian Youth, however the study was not without limitations which should be acknowledged in interpreting results. The small number of participants in the current study resulted in the failure to detect statistically significant differences on inferential analyses providing limited evidence. The nature of surfing and more generally outdoor sports creates challenges for recruitment and data collection as they are often enjoyed individually or in small groups in inaccessible environments. This is a common affliction associated with studying sports conducted in nature such as adventure and extreme sports (Feletti and Brymer, 2018) and explains the retrospective nature of many studies in this area. Additionally, the use of a single cohort of participants from one region in Australia may have limited the generalisability of the current findings as experiences of surf therapy may differ among youth from other regions. Finally, the daily stressors associated with the global pandemic COVID-19 may have confounded the findings.

## Implications and Future Directions

The current study represents a small-scale study which demonstrates the feasibility of surf therapy as an effective intervention for Australian at-risk youth. In line with previous research, preliminary findings from this study indicate that surf therapy may be effective in reducing depressive symptoms and increasing self-esteem, resilience, and social connectedness among adolescents. Moreover, this study uniquely contributes to the current evidence-base by examining these effects in an Australian context thus providing a foundation for future research in this area. Subsequently, taking into considerations the limitations of the current study, future research should endeavour to replicate the current study with a large sample utilising multiple cohorts to increase statistical power and generalisability of findings. Furthermore, future research endeavours may wish to recruit a more homogeneous sample to better determine the impact of the surf therapy program. Where possible, utilising artificial surf/wave pools may also strengthen future research by facilitating greater control of the surfing conditions (i.e., controlling wave height and frequency).

## Conclusion

The current study was the first to utilise a mixed method approach to examine the impact of surf therapy on at-risk youth in Australia. The findings add to the growing body of literature on surf therapy, by allowing for a unique understanding of participants experiences in the Australian context. In line with the existing literature, results indicated that an 8 week surf therapy program may increase self-esteem, resilience, and social connection, and reduce depressive symptoms among Australian adolescents by facilitating a shared experience in a unique learning environment and fostering personal growth. Congruent with the EDP model (Brymer and Davids, 2013) the unique benefits of surf therapy can be attributed to the individual-environment interaction as surfing allows for the immersion and engagement of the individual mediated by the unique affordances of nature. Moreover, these findings provide preliminary evidence of the efficacy of surf therapy among Australian youth and offer a potential starting point for further research utilising larger more diverse samples.

## Data Availability Statement

The raw data supporting the conclusions of this article will be made available by the authors, without undue reservation.

## Ethics Statement

The studies involving human participants were reviewed and approved by Navitas Professional Institute Human Research Ethics Committee. Written informed consent to participate in this study was provided by the participants' legal guardian/next of kin.

## Author Contributions

RM, TC, and KN-P contributed to conception and design of the study. RM and JP organised the data collection, with assistance from PW. RM and TC performed the data analysis. RM wrote the first draft of the manuscript. TC wrote sections of the manuscript. All authors contributed to manuscript revision, read, and approved the submitted version.

## Conflict of Interest

The authors declare that the research was conducted in the absence of any commercial or financial relationships that could be construed as a potential conflict of interest.

## Publisher's Note

All claims expressed in this article are solely those of the authors and do not necessarily represent those of their affiliated organizations, or those of the publisher, the editors and the reviewers. Any product that may be evaluated in this article, or claim that may be made by its manufacturer, is not guaranteed or endorsed by the publisher.
